# Multi-Platform Detection of MMP-7 in Colorectal Carcinoma

**DOI:** 10.3390/cancers18020214

**Published:** 2026-01-09

**Authors:** Ivana Večurkovská, Marek Stupák, Jana Kaťuchová, Veronika Roškovičová, Martin Pavluš, Mária Mareková, Jana Mašlanková

**Affiliations:** 1 Department of Medical and Clinical Biochemistry, Faculty of Medicine, Pavol Jozef Šafárik University in Košice, Trieda SNP 1, 040 11 Košice, Slovakia; ivana.vecurkovska@upjs.sk (I.V.); marek.stupak@upjs.sk (M.S.); maria.marekova@upjs.sk (M.M.); 2 Department of Technical Disciplines in Health Care, Faculty of Health Care, University of Presov, Partizánska 1, 080 01 Prešov, Slovakia; 3 1st Department of Surgery, Faculty of Medicine, Pavol Jozef Šafárik University in Košice, Trieda SNP 1, 040 11 Košice, Slovakia; jana.katuchova@upjs.sk (J.K.);; 4 Department of Transplant Surgery, F.D. Roosevelt Hospital Banská Bystrica, Nám. L. Svobodu 1, 975 17 Banská Bystrica, Slovakia

**Keywords:** MMP-7, colorectal cancer, biomarker, tumor progression

## Abstract

Background/Objectives: Matrix metalloproteinase-7 (MMP-7) has been implicated in colorectal cancer (CRC) progression; however, its relationship to disease stage and its suitability as a circulating biomarker remain unclear. This study aimed to determine whether MMP-7 expression and activity differ between benign and malignant colorectal conditions and whether serum MMP-7 levels reflect disease progression. Methods: mRNA MMP-7 expression data and MMP-7 levels have been collected from Gepia, Protein Atlas and UALCAN databases. For the study of patient samples, ELISA, Western blot, and zymography were used. The study included 30 patients with benign findings and 60 patients with colorectal cancer. The Gepia database reported significantly higher MMP-7 levels in patients with CRC. Results: The Protein Atlas and UALCAN highlight a notable difference between benign and malignant colon adenocarcinoma patients. The MMP-7 level in tissue samples from the malignant group, evaluated by Western blot, was approximately 4.5 times higher than in the benign group, and almost 3 times higher in serum samples. Using zymography, patients in the malignant group had MMP-7 activity more than 4x higher than that of patients in the benign group. The ELISA results supported this increase in MMP-7 levels. The average MMP-7 level in the malignant group was 1.2-fold that in benign tissue samples and approximately 3-fold that in serum samples. Notably, significant sex-related differences in serum MMP-7 concentrations were observed, indicating that gender may influence the interpretation of this biomarker. Conclusions: The discordance between stable MMP7 mRNA expression and declining serum MMP-7 protein levels in advanced CRC suggests complex post-transcriptional and post-translational regulation of MMP-7 during disease progression. Although this finding contrasts with much of the existing literature, it should be regarded as novel and hypothesis-generating. These results indicate that serum MMP-7 may reflect early tumor-associated processes rather than late-stage tumor burden, warranting further investigation in larger, stage-stratified and longitudinal cohorts.

## Simple Summary

MMP-7 is considered a potential biomarker for colorectal cancer, but its clinical value remains uncertain. Using public databases and patient samples, we found significantly higher MMP-7 levels in colorectal cancer compared with benign conditions. Serum MMP-7 levels differed between men and women, highlighting the need for sex-specific interpretation. Contrary to expectations, serum MMP-7 levels decreased with advancing disease stages, suggesting that MMP-7 may be more suitable for early cancer detection than for monitoring tumor burden.

## 1. Introduction

Colorectal carcinoma (CRC) is widespread, particularly in some regions of Europe and the Americas; however, the highest incidence is observed in Australia/New Zealand. Mortality rates are highest in Eastern Europe. CRC ranks second in incidence and third in cancer-related mortality worldwide [1]. Diagnosis, prediction, and correct treatment adjustment for CRC patients are complicated. Non-specificity or the total absence of symptoms often leads to late diagnosis [2]. Traditionally used screening methods have low sensitivity for early-stage CRC (guaiac-based fecal occult blood tests ≈ 50%, fecal immunochemical tests 18–33%) and are underused, especially in high-risk individuals [3]. An aggressive CRC tumor rapidly expands into vital organs, and its resection becomes impossible. Multiple possible mutations in CRC also complicate treatment efficacy [4,5,6]. Implementing new biochemical markers into clinical practice is a current challenge and a high priority for many research teams.

Metastasis of colorectal cancer is the last step in tumor progression and a significant cause of morbidity and mortality. It involves the spread of cancer cells from the primary tumor to nearby tissues and distant organs. The spread is mediated by complex molecular changes in cell-cycle regulation that alter the morphology and functions of epithelial cells, i.e., epithelial–mesenchymal transition (EMT). EMT involves disruption of intercellular relationships and adhesive properties of the cell matrix, breakdown of the extracellular matrix (ECM), and cleavage of basement membrane components by modulating matrix metalloproteinase (MMP) activity [7].

Matrix metalloproteinases (MMPs) are zinc-dependent endopeptidases involved in the remodelling of extracellular matrix (ECM), tumor invasion, and metastasis. MMPs degrade the basement membrane and ECM, allowing tumor cells to proliferate. Several MMP subtypes, including MMP-2, MMP-7, MMP-9, MMP-11, and MMP-14, have been identified as potential biomarkers for early detection, progression, and metastasis of colorectal cancer [8,9,10,11].

Unlike many matrix metalloproteinases, which are predominantly expressed by stromal cells, MMP-7 is mainly produced by epithelial tumor cells and plays a direct role in extracellular matrix remodeling, cell–cell interactions, and tumor–host signaling [12,13]. This epithelial origin distinguishes MMP-7 from other members of the MMP family. It links it directly to epithelial–mesenchymal transition (EMT), a key process in colorectal cancer progression characterized by loss of epithelial integrity and increased invasiveness.

MMP-7 is the smallest of all MMPs (known as matrilysin) and, compared to the others, lacks a C-terminal domain. MMP-7 is expressed by glandular and mucosal endothelial cells, keratinocytes, fibroblasts, and macrophages [14]. Inactive pro-MMP-7 (28 kDa) is converted into its active form (19 kDa) predominantly in response to oxidative and nitrosative stress-induced activation [13]. MMP-7 degrades multiple extracellular matrix and basement membrane components, including fibronectin, collagen type IV [15], laminin, and elastin, and directly cleaves E-cadherin, thereby promoting EMT-associated loss of epithelial integrity and invasion in colorectal cancer [16,17,18,19]. In addition, MMP-7 has been shown to inhibit apoptosis by proteolytically processing Fas ligand in intestinal and colorectal tumor models [20], and to promote tumor angiogenesis by releasing matrix-bound pro-angiogenic factors, with expression levels correlating with micro-vessel density and poor prognosis in CRC patients [20,21].

Thus, MMP-7 plays an essential role in regulating several processes, including ageing, wound healing, bone growth and remodelling, and signalling pathways that control cell growth, inflammation, and angiogenesis [22].

An upregulated MMP-7 level is closely associated with tumor growth, migration, and metastasis in CRC. Several studies have shown that the level of MMP-7 is significantly higher in serum and tissue samples of patients with colorectal cancer than in healthy controls [12,23,24]. The 5-year overall survival was significantly lower in patients with high serum MMP-7 levels than in those with lower levels in the control group. Therefore, MMP-7 appears to be an independent prognostic indicator of overall survival in CRC [14].

In colorectal cancer, MMP-7 has been implicated in early tumorigenesis, invasion, and modulation of the tumor microenvironment, and it has been proposed as a circulating biomarker. However, published data on MMP-7 expression across disease stages remain inconsistent, particularly when comparing transcript levels with protein abundance and enzymatic activity. This discrepancy is likely attributable to the complex post-transcriptional and post-translational regulation of MMP-7, which is especially relevant in EMT-associated tumor remodeling. Together, these factors provide a strong rationale for a multi-platform analytical approach that integrates with transcriptomic databases and complements protein-level and activity-based assays [12,14,22,23,24]. Finally, several ongoing and completed clinical trials registered in the ClinicalTrials.gov database have investigated MMP-7 as a diagnostic or prognostic biomarker in colorectal cancer (Table 1), further underscoring its clinical relevance.

## 2. Materials and Methods

### 2.1. Patients

The analysed group consisted of 90 patients who were hospitalised at the 1st surgical clinic of the Louis Pasteur University Hospital (UNLP) in Košice (Slovakia) and underwent surgery (biopsy/resection) because of suspected abnormalities of the colon and/or rectal tissue. This study was done with the ethics board’s concurrence (2020/EK/06042) and with the informed consent of all subjects. The research sample was divided into two subgroups based on histological findings: the control group with benign findings (BTG; 30 patients) and the research group with malignant findings (MTG; 60 patients). The control group included patients with diverticulitis, hemorrhoids, and adenomas. These conditions were selected because they represent common indications for colonoscopy or surgical intervention and are frequently encountered in routine clinical practice when colorectal cancer is suspected. Patients with inflammatory bowel disease, previous malignancies, or those who had undergone neoadjuvant therapy before sample collection were excluded from the study. The patient group was classified into the first (12 patients), second (24 patients), and third (24 patients) stages according to the standard TNM classification (Table 2).

### 2.2. Methods

Processing of biological materials (serum, tissue) and subsequent analyses were conducted at the Department of Medical and Clinical Biochemistry of the Faculty of Medicine, University of Pavol Jozef Šafárik (UPJŠ) in Košice. Serum samples were collected into tubes containing a separation gel (BD Vacutainer) and centrifuged at 3500 rpm for 5 min. The supernatant was aliquoted into Eppendorf microtubes and stored at −80 °C. The total protein concentration in the samples was measured using the Bradford method (absorbance at 595 nm).

Tissue samples were separated into 100 mg pieces and cleared of blood with PBS. 500 µL of commercial extraction buffer (T-PER Tissue Protein Extraction Reagent, ThermoFisher, Waltham, MA, USA) was added to 100 mg of tissue. After 20 min of incubation on ice, the tissues in the buffer were homogenized with a mechanical homogenizer. Tissue homogenates were centrifuged at 18,000 rpm for 20 min at 4 °C. The supernatant was aliquoted into 250 µL Eppendorf microtubes and stored at −80 °C. The total protein concentration in the samples was measured using the Bradford method. An experienced pathologist reviewed the tissue samples before analysis. Only regions with a high tumor cells proportion were selected for analysis. Areas with extensive necrosis or predominant stromal or inflammatory infiltration were excluded.

Samples were processed as rapidly as possible after collection to minimize proteolytic degradation. Samples were aliquoted to minimize freeze–thaw cycles, and each aliquot was thawed only once before analysis.

The ELISA method was used to quantitatively analyze MMP-7 (28 kDa; Human MMP-7 ELISA Kit; ThermoFisher; EH328RB). The ELISA kit used in this study is designed to detect total MMP-7 and does not distinguish between the pro-enzyme and active forms. Absorbance was measured using an Elx808 Microplate Reader (Biotech, Bratislava, Slovakia) at 450 nm, and the concentration was calculated from the standard curve. MMP-7 concentrations were expressed in ng/mL.

To confirm ELISA results, samples were analyzed by Western blot, run on a 4–12% polyacrylamide gel at 150 V. Protein samples were prepared in the presence of protease inhibitors to prevent protein degradation before downstream analyses. After electrophoresis, proteins were transferred from the gel onto a nitrocellulose membrane using a semi-dry transfer system at 15 V. The membrane was then blocked in 5% non-fat dry milk prepared in Tris-buffered saline with 0.1% Tween-20 (TBST) for 1 h at room temperature to prevent nonspecific binding. Subsequently, the membrane was incubated overnight at 4 °C with a primary anti-MMP-7 antibody (ab207299; Abcam, Cambridge, UK) diluted in blocking buffer.

After primary antibody incubation, the membrane was washed three times with TBST and incubated with a horseradish peroxidase (HRP)-conjugated secondary antibody (mouse anti-rabbit IgG monoclonal antibody, L27A9; Cell Signaling Technology, Danvers, MA, USA) for 1 h at 37 °C. Following additional washes with TBST, immunoreactive bands were visualized using enhanced chemiluminescence substrate (Pierce™ ECL Western Blotting Substrate; Thermo Fisher Scientific; Waltham, MA, USA, WJ335099) and detected with the iBright™ FL1500 Imaging System (Invitrogen, Waltham, MA, USA). MMP-7 was identified as a band at approximately 28 kDa.

To assess equal protein loading, membranes were stripped using a commercial stripping buffer according to the manufacturer’s instructions and re-probed with an anti-β-actin antibody. After incubation with the appropriate HRP-conjugated secondary antibody, β-actin signals were detected using the same chemiluminescent system. Densitometric analysis was performed, and MMP-7 signal intensities were normalized to β-actin for each sample.

For the analysis of MMP-7 active and inactive forms, gelatin zymography was performed on a 10% polyacrylamide gel copolymerized with 1 mg/mL gelatin at 150 V. After electrophoresis, the gel was washed in 2.5% Triton X-100 (2 × 30 min) and 100 mM Tris-base (2 × 5 min). Then, the gel was incubated at 37 °C in a renaturation solution containing Zn^2+^ and Ca^2+^ (24 h). After incubation, the gel was stained with 0.5% Coomassie Brilliant Blue G-250, Serva Electrophoresis GmbH, Heidelberg, Germany (1 h) and destained in a destaining solution (24 h). The enzymatic activity of individual MMP-7 forms was visible as white bands on the blue background. Individual MMPs were determined using a protein ladder (Spectra™ Multicolor Broad Range Protein Ladder; ThermoFisher; 26634). Protease inhibitors were used for protein quantification, but were omitted in zymography to preserve enzymatic activity.

Membranes from the Western blot and zymograms were evaluated in the ImageJ (Original) program (Laboratory for Optical and Computational Instrumentation, Madison, WI, USA (NIH & UW-Madison). The area data under the peaks was used to generate the graphs (as a relative amount). The term “area” refers to the integrated optical density (IOD) of each protein band, calculated as the product of the band intensity and the pixel area, using densitometric analysis in ImageJ. Western blot bands were normalised to β-actin as a loading control on the same membrane. Data are presented as relative, unitless values. Statistical analyses and graphical representations (Normality and log-normality tests, Mann–Whitney test, Kruskal–Wallis test, Dunn’s multiple comparison test, ROC curves) were performed using GraphPad Prism (version 8.0.1). Survival analyses were limited to Kaplan–Meier estimation, performed in IBM SPSS (version 29.0.1.0(171)). The data distribution was first evaluated using tests for normality (Shapiro–Wilk and log-normality). Since most datasets did not follow a Gaussian distribution, we employed non-parametric statistical methods, which are more appropriate for biological data with small sample sizes or heterogeneous variances. For comparisons between two independent groups, the Mann–Whitney U test was used; differences among more than two groups were assessed using the Kruskal–Wallis test followed by Dunn’s post hoc multiple comparison test. The diagnostic performance of individual biomarkers was further evaluated using receiver operating characteristic (ROC) curve analysis and calculation of the area under the curve (AUC). Patient survival was analyzed using the Kaplan–Meier method, which estimates time-to-event data while accounting for censored observations. To compare survival distributions between groups, we applied the log-rank (Mantel–Cox) test. In the graphs, we use the mean and standard deviation (SD) as error bars, which represent the variability or spread of the data around the mean.

Serum MMP-7 levels were measured once at baseline, before scheduled colorectal cancer (CRC) surgery, and tissue MMP-7 levels were measured from tissue collected at surgery. For survival analyses, the date of surgery was used as the starting point. Overall survival was defined as the time from surgery to death from any cause. The median follow-up time was 40 months. For Kaplan–Meier survival analyses, MMP-7 cut-off values were selected using a pragmatic, data-driven approach to ensure adequate group sizes and sufficient numbers of events in each group. No biologically predefined or statistically optimized threshold was applied. The log-rank test was used to assess differences between groups. Gene expression data for MMP-7 are publicly available in online gene databases. Our data are compiled and modified from http://gepia.cancer-pku.cn/. Consequently, these analyses were exploratory and intended to assess general survival trends rather than to define an optimal prognostic cut-off.

Multiple subgroup comparisons were performed without correction for multiple testing; therefore, the reported subgroup-specific associations should be considered exploratory and interpreted with caution, given the increased risk of false-positive findings.

## 3. Results

The relationship between gene expression and protein abundance in colorectal cancer is often complex and nonlinear. While transcriptomic analyses frequently serve as a first-line approach for biomarker discovery, accumulating evidence indicates that mRNA expression levels do not always accurately reflect protein concentration or enzymatic activity, particularly for proteases subject to extensive post-transcriptional and post-translational regulation. In the case of matrix metalloproteinase-7, publicly available transcriptomic datasets report relatively stable mRNA expression across colorectal cancer stages. In contrast, several protein-based studies suggest stage-dependent alterations in circulating proteins and enzymatic activity. This apparent discrepancy highlights the need to evaluate MMP-7 regulation beyond transcript abundance. It provides a rationale for integrative analyses combining transcriptomic data with complementary protein-level and activity-based assays.

### 3.1. Gene Expression of MMP-7 from the Database Gepia

**Figure 1:**
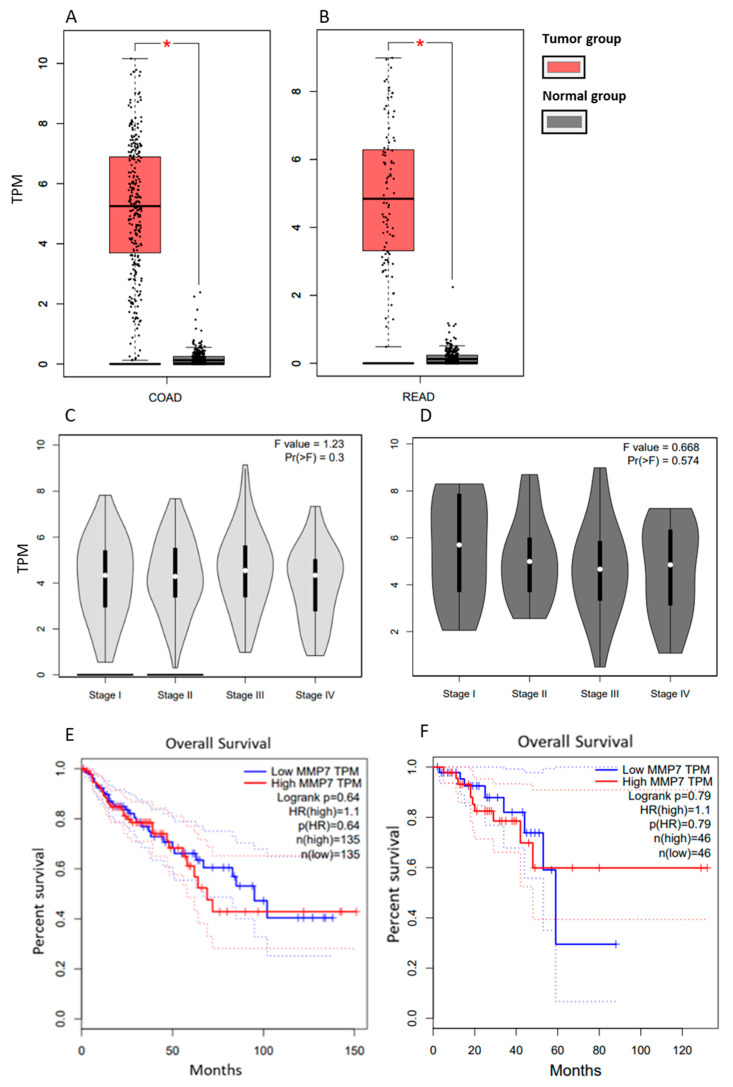
MMP-7 gene expression in tissue from patients with COAD compared to healthy controls (**A**) and in tissues from patients with READ compared to healthy controls (**B**) and MMP-7 gene expression in tissues of patients with COAD at different stages (**C**) and in tissues of patients with READ at different stages (**D**) Survival curves of COAD (**E**) and READ patients (**F**) based on MMP-7 gene expression. Modified according to the Gepia database. COAD: Colon Adenocarcinoma; READ: Rectum adenocarcinoma. * *p* < 0.05.

The Gepia database provides gene expression data for colon and rectal cancer separately. In both types of tumors, it can be seen that CRC patients in MTG had significantly higher MMP-7 expression compared to patients in the normal group (*p* < 0.05) (Figure 1A,B). Still, there is no statistically significant difference in MMP-7 expression across patients at different clinical stages of CRC (Figure 1C,D). According to the Gepia database, survival curves indicate that patients with high TPM (Transcripts Per Million) values have slightly shorter survival times than those with low TPM values. Still, no statistically significant difference was observed (Figure 1E,F).

### 3.2. Protein Level

#### 3.2.1. Results Obtained from Databases

**Figure 2:**
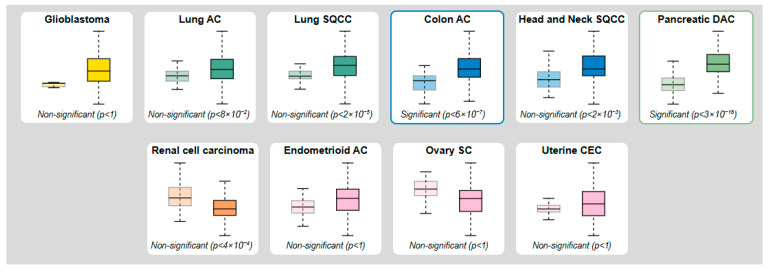
Protein levels of MMP-7 obtained by mass spectroscopy and modified according to the proteinatlas.org database (Left box—Normal tissue; Right box—Tumor tissue).

The proteinatlas.org database lists 10 tumor types with varying MMP-7 levels, based on tissue measurements obtained by mass spectrometry. In colon adenocarcinoma, the results show a significant difference between benign and malignant patients (*p* < 6 × 10^−7^) (Figure 2).

**Figure 3:**
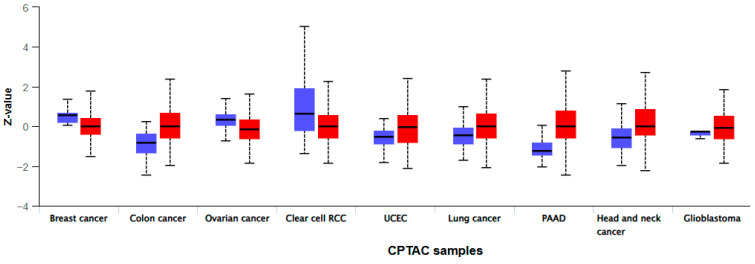
Protein levels of MMP-7 obtained by mass spectroscopy and modified according to the UALCAN database (Left box—Normal tissue; Right box—Tumor tissue).

The UALCAN database lists 9 tumor types with varying MMP-7 levels, based on tissue measurements obtained by mass spectrometry. In colon adenocarcinoma, the results show a significant difference between benign and malignant patients (*p* < 2 × 10^−12^) (Figure 3).

#### 3.2.2. Our Results of MMP-7 Levels by Western Blot

Since the results of the Protein Atlas database and ULCAN indicate significant differences between patients with malignant CRC and benign findings, we focused on protein techniques, ELISA, Western blot and zymography, which would confirm these results.

**Figure 4:**
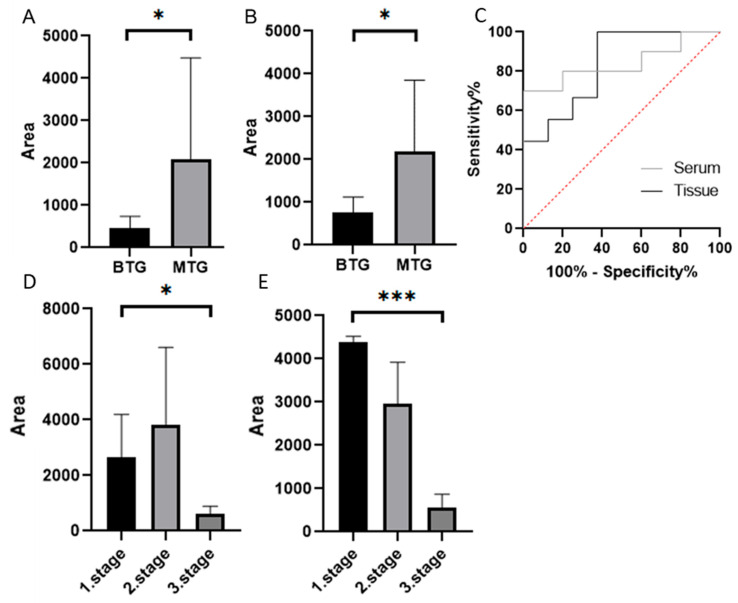
MMP-7 in tissue samples (**A**), and in serum samples (**B**) using Western blot (Mann–Whitney test *p* < 0.05 *), ROC curves (**C**). MMP-7 in individual stages of CRC in tissue (**D**) and serum samples (**E**) (Dunn’s multiple comparison test *p* < 0.05 *, *p* < 0.001 ***). Quantification represents densitometric analysis of band integrated optical density (IOD), normalised to β-actin (Supplement S2). BTG: Benign tumor group, MTG: Malignant tumor group.

The average MMP-7 area in the tissue samples of the BTG group was 463.67. In the MTG group, it was 2063.11 (Figure 4A). In the serum samples, in the BTG group, the average MMP-7 area was 770.61. In the MTG group, it was 2179.49 (Figure 4B). The Mann–Whitney test confirmed a statistically significant difference between groups (*p* < 0.05) for both sample types. ROC curve analysis (Figure 4C) demonstrated measurable sensitivity and specificity for distinguishing the BTG and MTG groups in tissue and serum samples. However, the results should be interpreted with caution, given the limited cohort size (Supplement S1).

The MTG was divided into subgroups based on CRC stage. The highest MMP-7 level in tissue samples was observed in the second stage of CRC (3834.18). The lowest value was observed in the third stage of CRC (601.87) (Figure 4D). The lowest MMP-7 level in serum samples was also observed in the third stage of CRC (557.19). The highest level was observed in the first stage of CRC (4326.21) (Figure 4E). The Kruskal–Wallis test confirmed statistically significant differences between groups in both tissue samples (*p* < 0.05) and serum samples (*p* < 0.001). Dunn’s multiple comparison test revealed the most significant difference between patients in the first and third stages of CRC in serum samples. We did not report ROC curves due to the small number of patients per group, which could compromise the reliability and interpretation of the results.

#### 3.2.3. Our Results of MMP-7 Levels by Zymography

**Figure 5:**
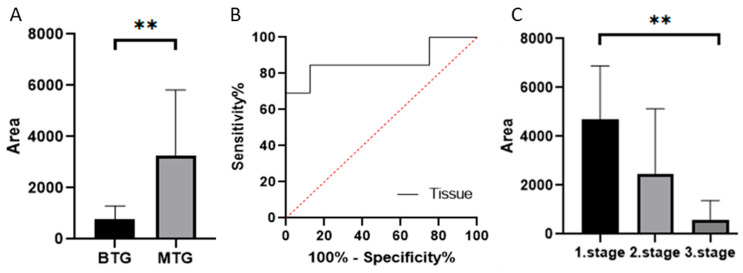
Relative abundances/peak areas of active forms of MMP-7 obtained by zymography ((**A**) Mann–Whitney test *p* < 0.01 **, (**C**) Dunn’s multiple comparison test *p* < 0.01 **). ROC curve comparing BTG and MTG groups based on MMP-7 levels (**B**) Quantification represents densitometric analysis of band integrated optical density (IOD), normalised to β-actin. BTG: Benign tumor group, MTG: Malignant tumor group.

Gelatin zymography was used to determine the levels of active MMP-7 (19 kDa), which was 788.08 in patients in the BTG group and 3277.71 in patients in the MTG group. The Mann–Whitney test confirmed a statistically significant difference between groups (*p* < 0.005) (Figure 5A). Based on ROC curves (Figure 5B), the distinction between BTG and MTG groups in tissue samples was found to be sensitive and specific (*p* = 0.0059). ROC curve analysis suggested that MMP-7 has discriminatory capability between BTG and MTG groups in both tissue and serum samples; however, validation in larger cohorts is required (Supplement S3).

After dividing the MTG by CRC stage, levels of active MMP-7 decreased with increasing stage (4691.49, 2457.21, and 938.82, respectively). The Kruskal–Wallis test confirmed a statistically significant difference between groups (*p* < 0.005) (Figure 5C).

**Figure 6:**
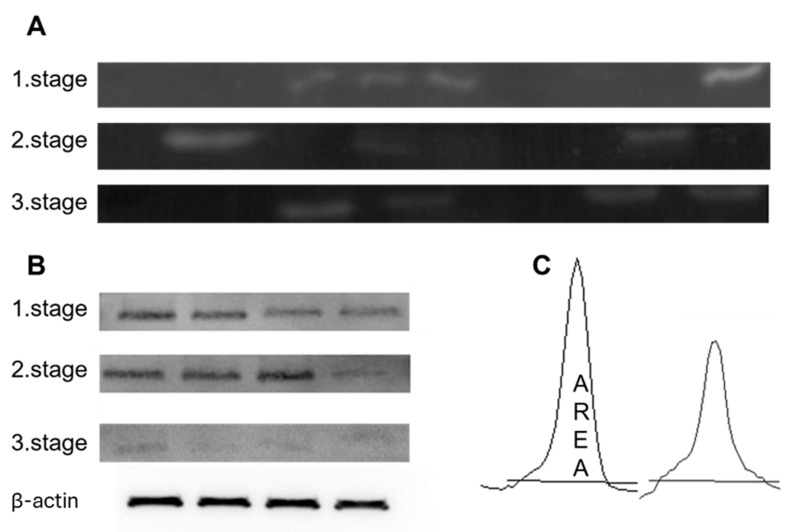
Representative figures of the results obtained by zymography (**A**) and by Western blot (**B**). All bands were analysed for peak areas (**C**) using ImageJ. (**B**) Representative cropped Western blot images are shown. β-actin was used as a loading control and was detected on the same membrane for normalization of protein expression. The uncropped blots are shown in Supplement S3.

In Figure 6, we have attached the cut-out gels from zymography (A) and Western blotting (B), and an example band in ImageJ from which we calculated intensities.

#### 3.2.4. Our ELISA Results of MMP7 Levels

**Figure 7:**
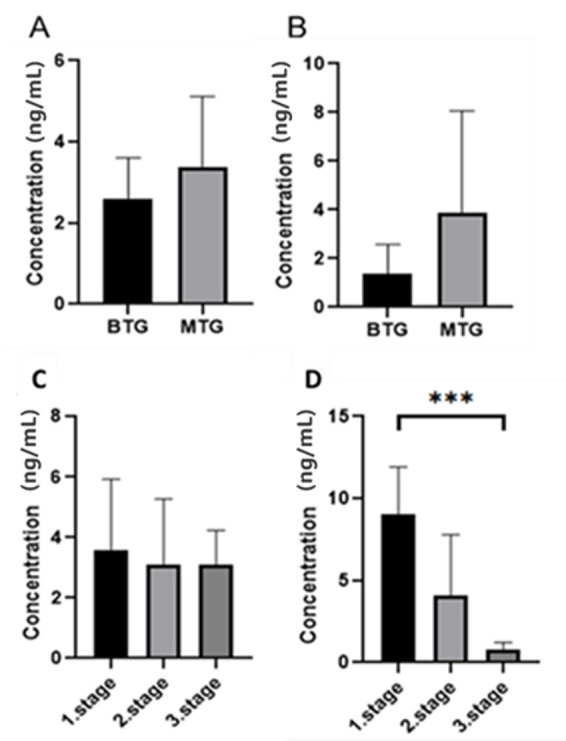
Results of MMP-7 concentration determination between malignant and benign groups in tissue ((**A**) Mann–Whitney test *p* > 0.05) and serum (**B**) Mann–Whitney test *p* > 0.05) and between individual stages of the malignant group of patients in tissue ((**C**) Dunn’s multiple comparison test *p* > 0.05) and serum ((**D**) Dunn’s multiple comparison test *p* < 0.005 ***).

The ELISA kit used in this study is designed to detect total MMP-7 and does not distinguish between the proenzyme and active forms, as with zymography. We agree that this represents a limitation of the ELISA-based quantification. ELISA measurements confirmed that patients in the BTG group had lower levels of MMP-7 than patients in the MTG group in both tissue samples (2.77 ng/mL versus 3.31 ng/mL) and serum (1.42 ng/mL versus 4.07 ng/mL) (Figure 7), but a statistically significant difference was not confirmed. After dividing the MTG by CRC stage, we found that serum MMP-7 levels decreased with increasing stage (9.01, 4.11, 0.82, respectively). The statistically significant difference was confirmed (*p* < 0.001) (Figure 7). In tissue samples, we did not observe a significant decrease (3.59, 3.08, 3.09, respectively). The results of MMP-7 levels, determined in serum by ELISA, show substantial differences between females and males, between living and deceased patients, and across individual stages (Table 3). The results of MMP-7 levels measured in tissue by ELISA show significant differences only between age groups, those under 60 years and those over 60 years. After careful consideration, we agree that a comprehensive ROC analysis, including AUC, sensitivity, specificity, and 95% confidence intervals, would require a larger and more balanced cohort of patients to ensure statistical robustness and a reliable estimate of diagnostic accuracy.

Therefore, given the relatively limited sample size and stage stratification in this study, we chose not to present diagnostic performance metrics based on ROC curves, as they could be potentially misleading or overinterpreted.

We evaluated serum and plasma MMP-7 levels obtained at diagnosis and used these baseline values to stratify patients in the Kaplan–Meier survival analysis. Since there is no internationally validated cut-off value for serum MMP-7 in colorectal cancer, we needed to define a threshold in our cohort. We therefore chose a data-driven approach that enabled us to create patient groups comparable in size and amenable to statistical analysis. We realize that the cut-off value determined in this way may not be universally applicable and requires confirmation in independent cohorts. Therefore, we interpret our results as preliminary exploratory evidence of the prognostic significance of MMP-7, while also emphasising the need for further validation.

**Figure 8:**
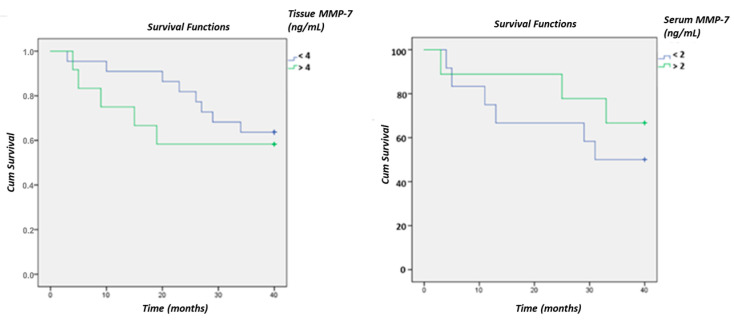
Survival curves of CRC patients based on MMP-7 levels in tissue and serum using the ELISA method.

A significant difference in MMP-7 between living and deceased patients was not confirmed using Kaplan–Meier curves, although a clear difference is evident, especially in tissue samples (Figure 8).

## 4. Discussion

The observation that MMP7 mRNA levels remain relatively stable across tumor stages in the GEPIA database, while our results indicate that MMP-7 protein concentrations decrease in later disease stages, highlights a potentially complex, non-linear regulation of MMP-7 during colorectal cancer progression. Although elevated matrix metalloproteinase expression is commonly associated with advanced and metastatic disease, this discordance between transcript and protein levels suggests that post-transcriptional and post-translational mechanisms may strongly influence MMP-7 regulation in colorectal cancer. MMP-7 expression and activity are subject to multiple layers of control, including regulation of translation efficiency, microRNA-mediated repression, mRNA stability governed by RNA-binding proteins, post-translational modifications, conversion from the pro-enzyme to the active form, sequestration through binding to the cell surface or extracellular matrix, and inhibition by tissue inhibitors of metalloproteinases (TIMPs). Collectively, these mechanisms may result in stable mRNA expression despite reduced circulating or detectable protein levels in advanced disease stages. While speculative, this regulatory complexity may partially explain the apparent discrepancy with reports linking MMP-7 to tumor progression and underscores the need for further studies to delineate stage-specific control of MMP-7 expression and activity in colorectal cancer [25,26].

It is necessary to consider changes in the ratio of protein synthesis to degradation as the disease progresses. In advanced stages, there may be increased extracellular degradation of MMP-7, increased TIMP inhibition, or increased antibody-mediated removal of MMP-7, resulting in lower detectable protein concentrations while maintaining relatively stable transcription. These regulatory elements are well described in the literature on MMP family regulation [25,27]. The differences between mRNA levels in homogenized tissue and released protein measured in tissue or serum during progression could also reflect changes in the origin of cells and the composition of the tumor (TME). With tumor growth and the development of metastatic stroma, epithelial tumor cells often undergo dedifferentiation, stromal populations undergo remodelling, and the phenotype of protease secretion changes. For example, if a larger proportion of stromal cells expressing a different MMP/TIMP profile predominates in later stages, the overall level of released MMP-7 may decrease, even though the mRNA measured in the whole sample does not change [13,28].

Finally, it cannot be ruled out that differences in measurement platforms contribute to the observed phenomenon: current transcriptomic analyses (RNA-seq/qPCR) measure total mRNA in tissue, while protein measurements (immunohistochemistry, ELISA, Western blot) are sensitive to protein form (pro-enzyme vs. active enzyme), localization (cell surface vs. secretory fraction), and the presence of inhibitors. Therefore, we recommend interpreting mRNA vs. protein differences as biologically interesting, not as an error, requiring targeted follow-up experiments (e.g., co-localization IHC + in situ hybridization, pro- vs. active form MMP-7 analyses, simultaneous TIMP and miRNA measurements).

Most research focuses on either serum or tissue MMP-7 levels. Our study compared both and revealed a stronger correlation between tissue MMP-7 and prognosis, as well as correlation between serum MMP-7 levels and tumor progression.

Some studies [9,29,30,31] have explored MMP-7 as a biomarker, our findings contribute to defining its clinical applicability in different disease stages.

Barabas et al. reported that serum MMP-7 was significantly correlated with advanced tumor stages (*p* < 0.05). Still, they concluded that it is also activated in premalignant adenomas, which cannot be confirmed as a biomarker of tumor progression [9].

Pesta et al. found statistically significant differences in MMP-7 mRNA levels between normal colorectal tissue and tumor tissue. They did not find any statistically significant correlations between MMP-7 mRNA levels and tumor localisation, clinical stage, or disease course. Based on these results, the clinical use of this approach for prognostication is ambiguous [30].

In the study by Wu et al., MMP7 protein expression was upregulated during CRC progression. They demonstrated that MMP-7 protein expression in CRC was positively associated with TNM stage, distant metastasis, and lymph node status, indicating that MMP-7 expression is closely related to CRC metastasis. In this study, however, one group comprised stages I and II, and the other comprised stages III and IV, which differed from our results, in which our groups comprised patients with only one stage of disease. In terms of survival, their results are consistent with ours. Survival analysis showed that increased MMP7 expression is associated with a poor prognosis in patients with CRC [31].

The prognostic role of serum MMP-7 in colorectal cancer is not uniformly established throughout the literature. The existing evidence is inconsistent rather than definitive, and to address this discrepancy, the present study aims to integrate transcriptomic data with protein-level and activity-based analyses in well-defined patient cohorts. Accordingly, our findings should be interpreted as hypothesis-generating and contributing to the ongoing discussion regarding the context-dependent prognostic value of serum MMP-7.

Our ROC analysis suggests a promising cut-off value for distinguishing CRC from benign conditions. The MMP-7 levels in tissue samples evaluated in the MTG group by the Western blot method were approximately 4.5 times higher than in the BTG group and almost 3 times higher in serum samples. Interestingly, MMP-7 levels in tissue were highest in Stage II CRC and lowest in Stage III. In serum samples, the highest MMP-7 level was observed in Stage I, while the lowest was in Stage III. These fluctuations suggest complex regulation of MMP-7 levels throughout CRC progression, possibly influenced by changes in the tumor microenvironment, immune system interactions, or extracellular matrix remodelling. The ROC curve analysis demonstrated high sensitivity and specificity, supporting MMP-7 as a potential biomarker for CRC diagnosis. We emphasize that future studies with larger, independent cohorts will be necessary to perform validated ROC analyses and establish clinically meaningful cut-off values.

ELISA results supported this increase in MMP-7 levels. An average of MMP-7 levels in MTG was 1.2 times higher than in BTG in tissue samples and approximately 3times higher in serum samples. This finding aligns with previous research suggesting that MMP-7 is upregulated in colorectal carcinoma (CRC) and plays a role in extracellular matrix degradation, tumor invasion, and metastasis. The absence of a significant difference between benign and malignant groups suggests limited diagnostic value of serum MMP-7. However, the observed stage-dependent decline in serum MMP-7 in the colorectal cancer cohort may reflect changes in tumor biology, systemic regulation, or protease compartmentalization during disease progression. Given the limited sample size and exploratory nature of this analysis, these findings should be considered hypothesis-generating and warrant validation in larger, stage-stratified cohorts.

Several studies suggest that single-nucleotide polymorphisms (SNPs) in the MMP-7 gene influence MMP-7 level and CRC susceptibility. Our results indicate high MMP-7 levels in early-stage CRC, which aligns with genetic studies showing that specific MMP-7 genotypes are associated with aggressive tumor behavior: Wu et al. found that the MMP-7-181A/G polymorphism increases the risk of CRC and enhances MMP-7 transcriptional activity, leading to greater tumor invasion and metastasis [32,33]. Zeng et al. reported an association between MMP-7 overexpression and epithelial-to-mesenchymal transition (EMT), suggesting that MMP-7 contributes to tumor invasiveness and stemness [34].

The findings complement our results showing a stage-dependent decrease in serum MMP-7, suggesting that MMP-7-driven tumor progression may depend on genetic predisposition and microenvironmental factors.

These results were also confirmed by Zhou et al. In his study, MMP-7 levels were not associated with sex, age, peritoneal dissemination, lymph node metastasis, or liver metastasis in CRC patients but was associated with histological grade, tumor size, TNM stage, and depth of tumor invasion. In addition, MMP-7 levels were significantly lower in chemotherapy-sensitive patients, who had markedly better overall survival than chemotherapy-resistant patients [35].

Our results suggest statistically significant differences in serum MMP-7 concentrations between women and men; however, these findings should be interpreted with caution. The sex-stratified analyses were based on relatively small subgroups and were not powered a priori to support definitive or causal conclusions. Accordingly, these observations should be regarded as exploratory and hypothesis-generating.

The biological mechanisms underlying higher MMP-7 levels in females are likely to be multi-layered [36,37]. MMP gene regulation by sex hormones is well documented. Estrogen signals change MMP transcription and are associated with the modulation of inflammatory pathways and ECM remodelling. This means that differences in endogenous estrogen levels (including postmenopausal status) or exogenous hormone therapy may influence MMP-7 level and its release into the circulation [38]. However, these mechanisms were not directly assessed in the present study.

Another potential mechanism for higher serum MMP-7 concentrations in women may involve gender differences in metabolism and systemic inflammation Prior studies indicate that colorectal cancer-associated metabolic and inflammatory profiles differ between men and women, potentially influencing signaling pathways involved in MMP-7 induction. Similarly, reported sexual dimorphisms in the tumor microenvironment—including immune infiltration, stromal composition, and mesenchymal cell activation—may be associated with differential expression or secretion of proteases such as MMP-7. These hypotheses remain speculative in the context of our data and warrant targeted investigation [39,40,41,42,43].

Taken together, our findings raise the possibility that MMP-7 may behave differently across sexes, suggesting that sex stratification could be considered in future evaluations of its biomarker performance, including threshold determination, ROC analyses, and prognostic modeling. Importantly, these implications should be validated in larger, adequately powered cohorts. Furthermore, although hormone-related mechanisms and tumor microenvironmental factors represent plausible explanatory pathways, mechanistic studies will be required before any causal interpretation can be made [44].

Future research should therefore focus on confirmatory, sex-stratified analyses in independent populations, as well as on integrative studies examining associations among serum MMP-7 levels, menopausal status, hormone replacement therapy use, estrogen receptor expression, and tumor microenvironment characteristics. Such studies will be essential to determine whether the observed sex-related patterns reflect actual biological differences or arise from confounding or sampling effects.

The results of our work suggest a promising prognostic role of MMP-7 in CRC progression, as Kaplan–Meier curves indicate a difference in MMP-7 levels between living and deceased patients in tissue samples, although the difference is not significant. This suggests that while MMP-7 levels may be associated with prognosis, larger sample sizes or longer follow-up periods may be required to establish a definitive correlation. Additionally, factors such as tumor heterogeneity, patient comorbidities, or treatment responses could have influenced survival outcomes, potentially masking statistical significance. However, we acknowledge that although Kaplan–Meier analysis using a data-driven cutoff facilitates visualization, it may reduce statistical power and introduce bias.

This MMP-7 role is also reported by Koskensalo et al. (2011) [45], in tissue samples. In their work, they described a significant correlation between high MMP-7 expression and poor prognosis during 5-year follow-up in CRC patients using immunohistochemical methods. However, in this case, the authors did not confirm a correlation between MMP-7 and TNM stages of CRC [45]. Given the several differences between our work and theirs, it is essential to identify the most significant similarities and use them in clinical practice. For example, we will need to focus more closely on the differences between males and females, as our results and those of Zhou et al. (2023) [35] indicate significant gender differences in MMP-7 levels [30].

Using zymography, patients in MTG had MMP-7 activity more than 4times higher than that of patients in BTG. Zymography results showed a significant increase in active MMP-7 in the MTG group compared with the BTG group. The serum results for patients in the MTG group matched the Western blot results, showing a decrease in MMP-7 levels with increasing stage.

Wang et al. (2005) suggested that MMP-7 cleaves pro-gelatinases (pro-MMP-2 and pro-MMP-9) into their active forms during ovarian cancer [46]. Building on these prior observations, we propose a stage-dependent conceptual model in which MMP-7 and MMP-9 may play distinct roles in colorectal cancer progression. In this model, elevated MMP-7 levels in early-stage CRC may be associated with activation of downstream gelatinases, whereas in more advanced stages, MMP-2, and particularly, MMP-9 may become the predominant effectors of extracellular matrix remodeling, invasion, angiogenesis, and metastatic spread.

This proposed stage-specific shift is consistent with our previous observations [47] which showed a more prominent association of MMP-9 with later CRC stages, including higher levels in living patients compared with deceased patients, particularly in stage II disease, when tumor penetration through the bowel wall occurs. However, it is essential to emphasize that the current study did not directly assess MMP activation, proteolytic cascades, or temporal switching between MMP-7 and MMP-9 activity. Therefore, the proposed MMP-7/MMP-9 “stage-switch” should be understood as a conceptual framework that integrates existing literature with observational findings, rather than as a demonstrated biological mechanism. Future longitudinal studies measuring active and inactive MMP forms across disease stages will be required to directly test this model and determine whether a functional transition from MMP-7–dominant to MMP-9–dominant activity occurs during colorectal cancer progression.

The role of MMP-7 in diagnosis and prognosis remains controversial, as MMP-7 levels are dynamic and influenced by several factors. MMP-7 is regulated through several signalling pathways, one of the most important being the TGF-β pathway. The activity of this pathway is anti-tumorigenic in the early stages (immune response, cell apoptosis) but becomes pro-tumorigenic in the advanced stages (induction of invasiveness and angiogenesis) [4]. It is also possible that MMP-7 is a double-edged sword, inhibiting disease progression in specific contexts and promoting it in others.

## 5. Conclusions

The difference between mRNA stability and declining MMP-7 protein levels in the late stages likely reflects a complex interplay among post-transcriptional mechanisms, TME alterations, protein modifications, and differences in degradation and secretion. Therefore, in future studies, we recommend parallel measurement of MMP7 mRNA and protein in micro-dissected tissue subpopulations, evaluation of the pro/active form of MMP-7 and TIMP profile, analysis of relevant miRNAs and RNA-binding proteins, and ex vivo experiments to monitor protein stability and secretion under varying TME conditions. Such approaches could clarify causal mechanisms and improve the translation of MMP-7 as a biomarker.

For more precise conclusions, it will be crucial to expand the patient cohort to stratify patients by tumor aggressiveness, metabolic changes, and, indeed, the patient’s genetic background, age, and associated diseases, which may also significantly influence MMP-7 levels in the tumor microenvironment. Our study demonstrates that serum MMP-7 levels differ substantially between men and women, underscoring the importance of accounting for sex as a biological variable in biomarker research. These findings suggest that unstratified use of MMP-7 in diagnostics or prognostic models may lead to misinterpretation and reduced clinical value. The observed differences likely arise from multiple mechanisms, including sex hormone regulation, metabolic and inflammatory pathways, and tumor microenvironment dimorphisms.

Accordingly, we propose that MMP-7 should be further evaluated as a sex-specific biomarker in colorectal cancer, with stratified cut-off values and tailored prognostic models. Future research should clarify the influence of estrogen signalling, menopausal status, and hormone replacement therapy on MMP-7 expression and secretion. Integrating sex-based analyses into both mechanistic studies and clinical validation efforts will be essential to harness the full potential of MMP-7 in personalized CRC diagnostics and therapy.

Last but not least, we must state that the benign comparator group comprised heterogeneous non-malignant colorectal conditions, which may differentially affect MMP-7 expression due to inflammatory or pre-neoplastic processes; this heterogeneity should be considered when interpreting comparisons with malignant samples.

The sample size of the present study limits the ability to draw definitive conclusions regarding the relationship between cancer progression and MMP-7 levels, particularly in the context of metastatic disease. While matrix metalloproteinases are widely associated with tumor progression in various solid tumors, our findings suggest that serum MMP-7 dynamics in colorectal cancer may differ, especially across disease stages. Therefore, our results should be interpreted as hypothesis-generating rather than definitive evidence of a causal relationship. Larger, stage-stratified and longitudinal studies will be required to clarify the role of MMP-7 in colorectal cancer progression and metastasis.

## Figures and Tables

**Table 1:** The clinical trials that focused on the diagnosis or prognosis of CRC by MMP-7.

Study Number	Name	Aim	Conclusions
**NCT03151759**	MMP-7 Modulation by Short and Long-Term Radiotherapy in Rectal Cancer Patients	Investigating the effect on MMP7 expression in patients with rectal cancer undergoing different regimens of neoadjuvant radiotherapy (RT)	50 Gy irradiation of rectal cancer gives less tumor activation of MMP7, whilst it is up-regulated by 25 Gy and surgery, regardless of RT
**NCT02047188**	Observation of Micro-vessels and Invasion in Early Colorectal Lesions by NBI	Clarifying the role of narrow-band imaging (NBI) in the prediction of invasion depth and the formation of lesion appearance under NBI	NBI is of excellent use in predicting invasion depth for early colorectal neoplasms, and positive expression of MMP-7 is associated with the appearance of capillary pattern type IIIB.
**NCT01570452**	Matrilysin Expression in Different Stages of Colorectal Tumors	Analyzing MMP7 in the bowel and lymph nodes of different tumor stages and evaluating its expression as a cancer biomarker	MMP7 increases with dysplasia and cancer disease stage in tumor tissue as well as in the regional lymph nodes. It may be used as a complement the investigation of suspected locally advanced cancer.
**NCT01276379**	Study Evaluating Biomarkers in Patients With Colorectal Cancer and Native KRAS Treated with Chemotherapy + Cetuximab	Validation of the biomarkers BRAF, IGF1P/MMP7 and PI3K-PTEN to predict PFS in patients with advanced and/or metastatic colorectal cancer with non-mutated KRAS treated with standard chemotherapy plus biweekly cetuximab as first-line therapy.	The co-expression of MMP7 and phosphorylated insulin growth factor receptor is associated with a worse prognosis in patients with WT KRAS mCRC treated with anti-EGFR.

**Table 2:** Classification of patients based on individual criteria.

	Malignant Tumor Group			Benign Tumor Group
	1. Stage	2. Stage	3. Stage	
**Number (%)**				
	12 (13)	24 (27)	24 (27)	30 (33)
**Age average**				
	67.3	66	66.1	64.3
**Gender N (%)**				
Males	7 (58)	16 (67)	14 (58)	14 (47)
Females	5 (42)	8 (33)	10 (42)	16 (53)
**Involvement**				
	No	No	Yes	No
**Finding N (%)**				
Hemorrhoids	-	-	-	4 (14)
Diverticulitis	-	-	-	5 (18)
Adenoma	-	-	-	19 (68)
Adenocarcinoma	12 (100)	24 (100)	24 (100)	-
**Survival N (%)**				
Living	9 (75)	15 (63)	11 (46)	27 (90%)
Deceased	3 (25)	9 (37)	13 (54)	3 (10%)

**Table 3:** Comparison of MMP-7 levels in the malignant group of CRC patients using the ELISA method.

	Tissue MMP-7 (ng/mL)	Serum MMP-7 (ng/mL)
Females	3.35 (1.93–8.7)	6.83 (1.47–11.8)
Males	3.65 (1.32–7.9)	1.91 (0.25–6.3)
*p*	0.236	**0.046 ^a^**
<60 years	4.95 (1.93–8.7)	2.03 (0.70–4.07)
≥60 years	2.89 (1.62–3.92)	3.84 (0.25–11.8)
*p*	**0.016**	0.542 ^a^
Living patients	3.29 (1.31–8.7)	1.73 (0.24–6.11)
Deceased patients	3.42 (1.62–7.90)	5.40 (1.47–11.8)
*p*	0.988	**0.048 ^a^**
1. stage	3.59 (2.08–4.07)	9.01 (6.29–11.8)
2. stage	3.08 (1.31–8.7)	4.11 (1.07–11.1)
3. stage	3.09 (0.82–3.8)	0.82 (0.24–1.01)
*p*	0.463	**0.0004 ^b^**
Low-grade	3.72 (1.33–8.7)	6.30 (0.71–11.8)
High-grade	2.85 (2.08–4.1)	3.02 (0.23–11.1)
*p*	0.181	0.692 ^a^
Rectum tumor	4.14 (1.62–8.7)	5.46 (0.69–11.8)
Colon tumor	3.09 (1.31–4.10)	2.62 (0.63–10.54)
*p*	0.557	0.301 ^a^

^a^ Mann–Whitney test; ^b^ Kruskal–Wallis test.

## Data Availability

The raw data used in the current study are available from the corresponding author upon reasonable request for non-commercial use.

## Supplementary Materials

The following supporting information can be downloaded at: https://www.mdpi.com/article/10.3390/cancers18020214/s1, Supplement S1: Data from ROC curves comparing BTG and MTG groups of CRC patients based on MMP-7 expression in tissue by the western blot method; Supplement S2: Data from ROC curves comparing BTG and MTG groups of CRC patients based on MMP-7 expression by the zymography method; Supplement S3: Original Western blot figures.
