# Supplementary material for: Multi-Platform Detection of MMP-7 in Colorectal Carcinoma
Source: Cancers (Basel). 2026 Jan 9;18(2):214. doi: 10.3390/cancers18020214 (PMC12839346; doi:10.3390/cancers18020214)

Supplement S1: Data from ROC curves comparing BTG and MTG groups of CRC patients based on MMP-7 expression in tissue by the western blot method

	Tissue	Serum
The area under the ROC curve	0.8333	0.8400
Std. Error	0.1006	0.1041
95% confidence interval	0.6362 to 1.000	0.6360 to 1.000
P value	**0.0209**	**0.0373**

Supplement S2: Data from ROC curves comparing BTG and MTG groups of CRC patients based on MMP-7 expression by the zymography method.

Tissue	
The area under the ROC curve	0.8979
Std. Error	0.0947
95% confidence interval	0.7905 to 1.000
P value	**0.0059**

Supplement S3: Original Western blot figures

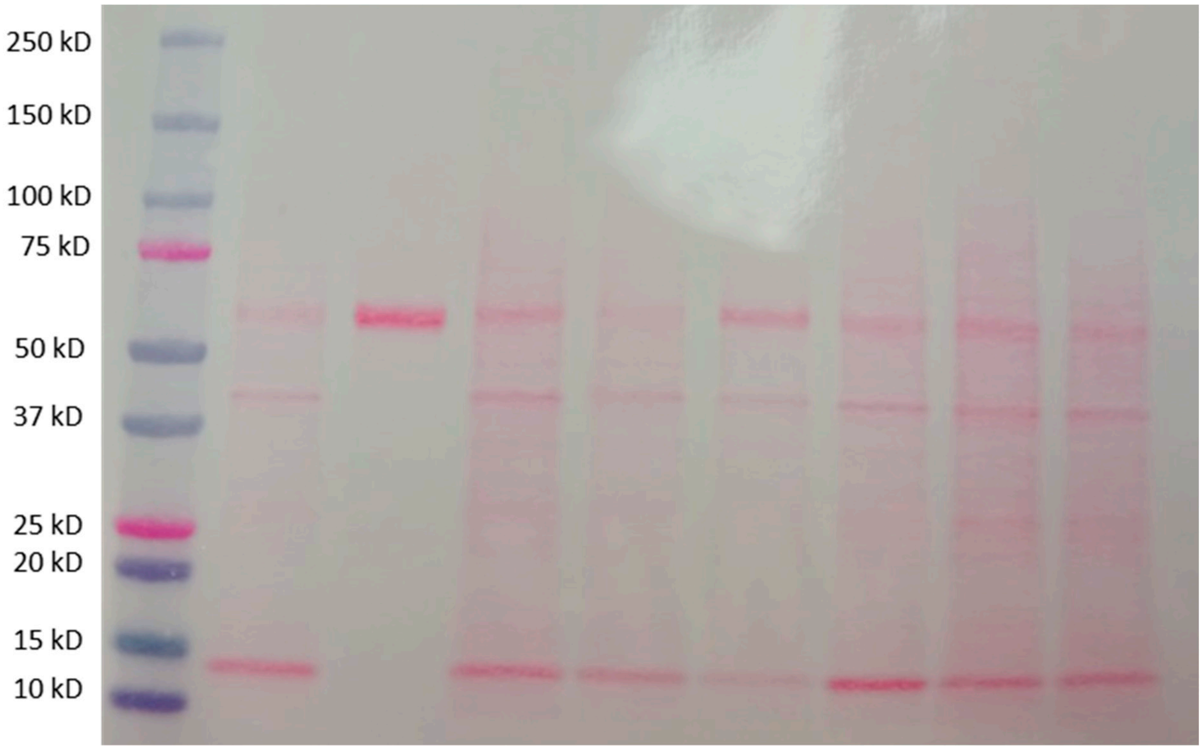

Supplement: Supplementary file 1 [file cancers-18-00214-s001.zip › cancers-4036409-supplementary.pdf]
